# Characterization of intrinsic subtypes of breast cancer and their relationship with staging: an observational study

**DOI:** 10.3389/fmed.2025.1553910

**Published:** 2025-05-09

**Authors:** Paola Tapia-Uriol, Lorena Becerra-Goicochea, Víctor Campos-Valderrama, Juana del Valle-Mendoza, Miguel Angel Aguilar-Luis, Wilmer Gianfranco Silva-Caso

**Affiliations:** ^1^Oncology Unit, Cajamarca Regional Teaching Hospital, Cajamarca, Peru; ^2^Professional Academic School of Obstetrics, National University of Cajamarca, Cajamarca, Peru; ^3^Biomedicine Laboratory, Research Center of the Faculty of Health Sciences, Universidad Peruana de Ciencias Aplicadas, Lima, Peru

**Keywords:** carcinoma, ductal, breast, breast neoplasm, breast carcinomas

## Abstract

**Background:**

Breast cancer is one of the main causes of morbidity and mortality among women around the world. In Peru, it has recently surpassed cervical cancer as the most commonly reported cancer. Studying the relationship between intrinsic breast cancer subtypes and disease staging can optimize diagnosis, prognosis, and treatment. Therefore, there is a need for better risk stratification, selection of personalized treatment, and improved early detection strategies. We conducted this study to address the lack of data on underrepresented populations such as the Peruvian population. The objective of the study was to analyze the distribution of intrinsic subtypes of breast cancer and their correlation with prognostic factors and demographic characteristics among women in Peru.

**Methods:**

A descriptive, retrospective observational study was conducted, analyzing 67 cases of breast cancer of various intrinsic subtypes diagnosed at a referral hospital in Peru. Clinical, demographic, and pathological data were collected, including histological type, intrinsic subtype, tumor stage, and geographic origin of the patients. Intrinsic subtypes were classified through immunohistochemistry, and the data were processed to determine their distribution and correlation with prognostic factors such as disease stage.

**Results:**

The mean age of the 67 patients included in the study was 54.2 years. The majority of cases originated from the city of Cajamarca (56.7%, *n* = 38). Invasive breast carcinoma of no special type was the most common histological type (62.7%, *n* = 42). Among the intrinsic subtypes, luminal B was the most common (31.3%, *n* = 21), followed by luminal A and triple-negative (22.4%, n = 15), both with the same frequency. Furthermore, 16.4% (*n* = 11) of patients presented with metastasis at the time of evaluation. A high frequency of tumors was observed in Tumor, Nodes, Metastasis (TNM) stages 3 and 4, accounting for 49.2% (*n* = 33).

**Conclusion:**

This study describes the heterogeneity of breast cancer based on the identification of intrinsic subtypes within the analyzed population. The high frequency of luminal B, luminal A, and triple-negative subtypes is notable. The highest frequency of identified cases was in the advanced stages, highlighting the need for personalized treatments and improved early detection strategies.

## Highlights

- The study highlights the heterogeneity of breast cancer within the analyzed population, identifying the intrinsic subtypes luminal A, triple-negative, and HER2-negative luminal B as the most common.
- The most frequent histological type was invasive breast carcinoma of no special type, observed in 61.2% of cases.

## Introduction

The Global Cancer Observatory (GLOBOCAN) reports that breast cancer is a public health threat in Latin America and the Caribbean, with 220,124 new cases (14.2% of all cancers) and 59,876 deaths (8% of cancer deaths) in 2022, second only to prostate cancer (14.6%) (1). The age-standardized incidence rate of female cancer is 177.4 cases per 100,000 women, and the mortality rate is 77.6 cases per 100,000 women. Moreover, GLOBOCAN (2022) reported the existence of 725,017 active cancer cases in the last 5 years, representing 17.7% of all cancer cases in the region (1, 2).

Breast cancer risk factors can be categorized as non-modifiable and modifiable, encompassing genetic, reproductive, and lifestyle factors. Among the non-modifiable factors, genetic mutations in the Breast Cancer Gene 1 and Breast Cancer Gene 2 (BRCA1 and BRCA2) are the most common alterations in breast cancer (3, 4). Among the modifiable factors, a study conducted in Argentina, Brazil, Chile, Mexico, and Uruguay showed differences in survival rates, access to healthcare, and socioeconomic context. These factors influence the outcomes, underscoring the need to consider geographic and cultural contexts in disease management (5). In Peru, breast cancer has surpassed cervical cancer as the most frequently reported cancer for the first time (6).

The Tumor, Nodes, Metastasis (TNM) staging system is used to determine the anatomical extent of malignant disease based on clinical and pathological criteria. It categorizes cancer into three categories: primary tumor (stage T), lymph node involvement (stage N), and metastasis (stage M). The TNM classification for breast cancer, described by the American Joint Committee on Cancer (AJCC), is periodically reviewed based on advances in diagnosis and treatment, but modifications are only introduced selectively to ensure its stability and reliability (7–9). It is also critical to identify the risk of post-treatment recurrence and to choose the appropriate adjuvant systemic therapy (10). However, the TNM system may not entirely capture the heterogeneity of breast cancer, and there are ongoing questions about the feasibility of using anatomic prognosis to apply systemic therapy (11).

According to current research (12), biological factors such as hormone receptors, human epidermal growth factor receptor 2 (HER2), and Ki-67 have a greater role to play in prognosis than TNM staging alone. HER2 is a key biomarker in breast cancer and is overexpressed in approximately 18% of cases, particularly in the HER2-enriched molecular subtype (13). As a result of the discovery of HER2, many anti-HER2 therapies have been investigated, focusing on personalized treatment approaches (14). Ki-67 is a proliferation marker used to categorize breast cancer subtypes and determine disease aggressiveness (15). These findings demonstrate that a deep understanding of cancer biology can improve the ability to predict outcomes and select optimal treatment options.

As a result, although TNM classification is critical for initial evaluation and planning, a comprehensive analysis of the biological subtypes of the tumor must be carried out in conjunction with this information (16). The integrated approach can help healthcare professionals tailor treatments based on the specific characteristics of each patient, which can improve their ability to achieve better health outcomes.

Gene expression analysis in breast cancer has revealed unprecedented diversity, both between and within tumors. Breast cancers are categorized into luminal A, luminal B, HER2-overexpressing (enriched), and triple-negative (basal-like) subtypes (17). Each subtype possesses unique genetic characteristics that influence clinical decision-making and treatment personalization. These subtypes differ in their histopathological and biological characteristics, which are responsible for varying responses to treatments and require different therapeutic strategies (18). Understanding of how cancer cells interact with their environment not only sheds light on their complex interactions but also provides insight into how these factors influence cancer evolution and clinical management (19). Treatment personalization based on the intricate biology of breast cancer, particularly through our understanding of intratumoral heterogeneity and organization, holds the potential to significantly improve treatment efficacy.

## Methods

### Study design

An observational, descriptive, correlational, and retrospective study was conducted on the distribution of intrinsic breast cancer subtypes and their relationship with demographic, clinicopathological, and prognostic factors at the Oncology Unit of the Regional Teaching Hospital of Cajamarca in 2023.

### Population and sample

The study population included all patients diagnosed with breast cancer in 2023. A non-probabilistic convenience sampling was used, including all patients who met the inclusion criteria. No predefined sample size was applied due to the importance of capturing all available cases for a comprehensive analysis, totaling 67 cases.

Inclusion criteria: Female patients diagnosed with breast cancer, those over 18 years of age, and those with complete demographic, histological, and staging data. Exclusion criteria: Female patients with a primary tumor of non-breast origin, those with breast or generalized infectious processes, those with incomplete data, or those who have refused any of the protocolized diagnostic and staging procedures.

### Data collection procedures

A standardized data collection sheet was developed and used by the research team to gather relevant information on intrinsic subtypes of breast cancer, TNM classification, and sociodemographic and clinical data. Data collection was based on reviewing medical records and institutional databases.

The breast pathology procedure was performed using a standardized protocol of the pathology department of the hospital where the study was performed, based on the recommendations given by Lester et al. (20). For each patient, a tumor slide and a paraffin block were selected for immunohistochemical analysis. The immunohistochemical study was performed according to the guidelines of the American Society of Clinical Oncology (ASCO)/College of American Pathologists (CAP) (21, 22). Estrogen receptor (ER) and progesterone receptor (PgR) were recorded as positive if staining was observed in more than 1% of cells. Her2/neu was considered positive if more than 10% of tumor cells stained completely and intensely, and “indeterminate” if more than 10% of tumor cells stained incompletely and weakly or if <10% stained completely and intensely. “Indeterminate” cells were tested by chromogenic *in situ* hybridization (CISH) to determine positivity. Ki67 was considered positive if it was expressed in more than 30% of cells (23, 24).

The immunohistochemical definition used was based on the 2015 St. Gallen International Expert Consensus, as described below (25, 26).

Luminal A: ER and/or PgR positive; HER2 negative; Ki67 low (<30%).

Luminal B: ER and/or PgR positive; HER2 negative; Ki67 high (≥30%) or.

ER and/or PgR positive; HER2 overexpressed or amplified; any Ki67.

HER2 enriched: ER and PgR absent; HER2 positive/overexpressed or amplified; any Ki67.

Triple-negative: ER and PgR absent; HER2 negative; any Ki67.

### Data analysis

A descriptive analysis was performed using absolute and relative frequencies, measures of central tendency, and dispersion for all patients (*n* = 67). All analyses were conducted using Stata/BE (18.0, StataCorp LLC, College Station, TX). The relationship between qualitative variables was examined using the Chi-squared test and Fisher’s exact test, as appropriate. For expected cell counts of <5, Fisher’s exact test was conducted. Quantitative variables were compared across more than two groups using analysis of variance (ANOVA) for normally distributed data, and the Kruskal–Wallis test for non-normally distributed data. Statistical significance was set at a two-sided *p*-value of 0.05.

### Ethical considerations

This study adhered to ethical principles respecting participants’ dignity, privacy, and autonomy, ensuring confidentiality and protection of personal data. Approval was obtained from the Ethics Committee of the Regional Teaching Hospital of Cajamarca, following relevant national and international biomedical research guidelines. The primary samples were collected as part of cancer epidemiological surveillance in Peru, which, due to public health mandates, does not require written informed consent. The present analysis was performed using a secondary database obtained from this epidemiological surveillance program.

## Results

The study included 67 women diagnosed with cancer in 2023. Table 1 summarizes the clinicopathological characteristics of the cases studied. The mean age of the cases was 54.2 years, with a minimum of 29 years and a maximum of 92 years. A predominance of cases in women over 50 years of age (64.2%, *n* = 43) was reported. Furthermore, 50% of the participants were over 52 years of age, and 25% were under 45 years of age. Regarding geographic location, 38 patients were from Cajamarca (56.7%), followed by 8 from Celendín (11.9%). Celendín is a semi-rural region with a population comparable to that of Cajamarca, but with more limited access to specialized health services.

**Table 1:** Clinicopathologic characteristics of breast cancer cases studied.

Clinicopathological characteristics	*N* = 67 (%)
Age (years)	
Mean ± SD	54.2 ± 13.2
Age groups	
< 50 years	24 (35.8)
≥ 50 years	43 (64.2)
Histologic type	
Invasive breast carcinoma of no special type (NST)	42 (62.7)
Invasive lobular carcinoma (ILC)	6 (8.9)
Others	19 (28.4)
Metastases	
No	40 (59.7)
Yes	11 (16.4)
ND	16 (23.9)
TNM stage	
Stage 1	1 (1.5)
Stage 2	17 (25.4)
Stage 3	21 (31.3)
Stage 4	12 (17.9)
ND	16 (23.9)

*Information not available (ND). Other histologic type categories include undifferentiated carcinomas and other rare carcinomas.

Regarding histological type, invasive breast carcinoma of no special type (NST) was detected in 42 of the 67 patients (62.7%), followed by 19 patients with other histological classifications (including undifferentiated carcinoma and other rare carcinomas), representing 28.4%, and invasive lobular carcinoma (ILC) in 6 patients (8.9%). The study revealed that the most common TNM stage was stage 3 (31.3%, *n* = 21), followed by stage 2 (25.4%, *n* = 17). Regarding lymph node extension, 26 patients were classified as N1 (38.8%), being the most common type. Furthermore, the absence of metastasis was observed in 40 cases (59.7%). The clinicopathological characteristics and frequencies of molecular subtypes of breast cancer are presented in Table 2. Among breast cancer subtypes, the distribution of histological type and TNM stage (including tumor stage, lymph node, and metastasis) was significantly different (*p* < 0.05). The relationship between clinicopathological characteristics and breast cancer subtypes was statistically significantly different for histological type (*p* = 0.003), primary tumor size (*p* = 0.011), regional lymph node involvement (p = 0.011), metastasis (*p* = 0.001), and TNM stage (p = 0.003). No differences were observed in the place of residence (*p* = 0.474) or age groups (*p* = 0.814) among the molecular subtypes of breast cancer (Table 2). Table 3 presents the frequency of breast cancer stages according to TNM staging. The most common subtypes were eight patients with luminal B subtype in TNM stage 4 (38.1%), four patients with luminal A subtype in TNM stage 2 (26.7%), and six patients with triple-negative subtype in TNM stages 2 and 3 (40% in each stage).

**Table 2:** General overview of the clinical and pathological characteristics of the molecular subtypes of breast cancer.

Clinical and pathological characteristics	**Subtypes of breast cancer**					
	Luminal A *N* = 15 (22.4%)	Luminal B *N* = 21 (31.3%)	HER2 enriched *N* = 5 (7.5%)	TNBC* *N* = 15 (22.4%)	Unknown *N* = 11 (16.4%)	*p*-value
Age (mean ± SD)	54.7 ± 15.8	50.8 ± 11.3	53.2 ± 9.4	57.2 ± 11.9	56.5 ± 16.4	0.648
Age groups						
< 50 years	41.0 ± 4.7	39.9 ± 6.7	38.0 ± 6.4	44.8 ± 5.4	42.4 ± 4.8	0.814
≥ 50 years	63.8 ± 13.8	57.5 ± 7.6	57.0 ± 4.7	61.7 ± 10.2	68.3 ± 12.2	
Histologic type						**0.003**
Invasive breast cancer of no special type (NST)*	8 (53.3)	17 (81.0)	3 (60.0)	11 (73.3)	3 (27.3)	
ILC*	3 (20.0)	3 (14.3)				
Others**	4 (26.7)	1 (4.8)	2 (40.0)	4 (26.7)	8 (72.7)	
Tumor						**0.011**
T1	2 (13.3)	1 (4.8)	–	1 (6.7)	–	
T2	5 (33.3)	5 (23.8)	1 (20.0)	5 (33.3)	1 (9.1)	
T3	2 (13.3)	6 (28.6)	1 (20.0)	5 (33.3)	2 (18.2)	
T4	1 (6.7)	8 (38.1)	2 (40.0)	3 (20.0)	-	
ND	5 (33.3)	1 (4.8)	1 (20.0)	1 (6.7)	8 (72.7)	
Lymph node extension						**0.011**
N0	3 (20.0)	4 (19.0)	1 (20.0)	4 (26.7)	2 (18.2)	
N1	6 (40.0)	11 (52.4)	1 (20.0)	7 (46.7)	1 (9.1)	
N2	1 (6.7)	3 (14.3)	2 (40.0)	3 (20.0)		
N3		2 (9.5)				
ND*	5 (33.3)	1 (4.8)	1 (20.0)	1 (6.7)	8 (72.7)	
Metastases						**0.001**
No	8 (53.3)	13 (61.9)	4 (80.0)	12 (80.0)	3 (27.3)	
Yes	2 (13.3)	7 (33.4)		2 (13.3)		
ND*	5 (33.3)	1 (4.8)	1 (20.0)	1 (6.7)	8 (72.7)	
TNM stage						**0.003**
Stage 1	1 (6.7)					
Stage 2	4 (26.7)	4 (19.0)	1 (20.0)	6 (40.0)	2 (18.2)	
Stage 3	3 (20.0)	8 (38.1)	3 (60.0)	6 (40.0)	1 (9.1)	
Stage 4	2 (13.3)	8 (38.1)		2 (13.3)		
ND*	5 (33.3)	1 (4.8)	1 (20.0)	1 (6.7)	8 (72.7)	
Location of residence						0.474
Bambamarca				1 (6.7)		
Cajabamba				2 (13.3)	2 (18.2)	
Cajamarca	8 (53.3)	13 (61.9)	3 (60.0)	8 (53.3)	6 (54.5)	
Celendin	4 (26.7)	1 (4.8)	1 (20.0)	1 (6.7)	1 (9.1)	
Chota		2 (9.5)		1 (6.7)		
Contumazá	1 (6.7)		1 (20.0)			
Cutervo		1 (4.8)		1 (6.7)		
Encañada		1 (4.8)				
San Marcos		2 (9.5)				
San Miguel	1 (6.7)					
San Pablo	1 (6.7)			1 (6.7)	2 (18.2)	
Santa Cruz		1 (4.8)				

*TNBC, Triple-negative breast cancer; NST, invasive breast carcinoma of no special type; ILC, invasive lobular carcinoma; ND, information not available. Other tumor categories include undifferentiated carcinoma and other rare carcinomas of the breast. Statistical significance is indicated by bolded *p*-values (<0.05). The chi-squared (*χ*^2^) and Fisher’s exact tests (for frequencies <5) were used. **Undifferentiated carcinoma and other rare carcinomas.

**Table 3:** Distribution of TNM stages in different breast cancer subtypes.

	**Subtypes of breast cancer**					
	**Luminal A** **N=15 (22.4%)**	**Luminal B N=21 (31.3%)**	**HER2** **enriched** **N=5 (7.5%)**	**TNBC** **N=15 (22.4%)**	**Unknown** **N=11 (16.4%)**	*p*-value*
TNM stage						**0.010**
I	1 (6.7)					
II A	4 (26.7)	3 (14.3)	1 (20.0)	3 (20.0)	1 (9.1)	
II B		1 (4.7)		3 (20.0)	1 (9.1)	
III A	2 (13.3)	3 (14.3)	1 (20.0)		1 (9.1)	
III B	1 (6.7)	3 (14.3)	2 (40.0)	3 (20.0)		
III C		2 (9.5)		3 (20.0)		
IV	2 (13.3)	8 (38.1)		2 (13.3)		
ND	5 (33.3)	1 (4.7)	1 (20.0)	1 (6.7)	8 (72.7)	

*Statistical significance is indicated by bolded *p*-value values (< 0.05). Chi-square (χ²) and Fisher’s exact tests (for frequencies < 5) were used.

## Discussion

The incidence of breast cancer in the population reflects a global upward trend. It has been reported that breast cancer has surpassed lung cancer in incidence and mortality, and the rates vary between developed and developing countries. The disparity in mortality rates highlighted by Sung et al. (2) highlights the importance of localized risk analysis and unified management strategies. Sharma’s study (27) reports a rising incidence of breast cancer in South America, with the highest rates in Uruguay, with an incidence of 72.65 per 100,000 persons and a mortality of 29.97 per 100,000 persons, and the lowest rates in Peru, with an incidence of 27.63 per 100,000 and a mortality of 10.79 per 100,000 persons. In their analysis, most South American countries recorded an increase of more than 70% in incident cases (27). The pattern described highlights the urgent need to improve cancer prevention and care systems in emerging regions with higher incidence and mortality.

In this context, our series reports a high incidence of invasive breast carcinoma of no special type (62.7%) in patients diagnosed with breast cancer with a mean age of 54.2 years, consistent with reports published to date (28). Invasive breast carcinoma of no special type is recognized as the most common form of breast cancer, accounting for approximately 70–80% of invasive breast cancer cases (29). The mean age at diagnosis in this study is also consistent with global series, which typically place breast cancer diagnosis in women approximately 50 years of age (30).

The observation that a significant proportion of patients are in advanced TNM stages 3 and 4 at the time of diagnosis is concerning and consistent with recent studies in regions with limited resources or precarious health systems, where late diagnosis remains a persistent problem (31). This trend highlights the need to improve early detection strategies. For example, studies conducted in developed countries indicate that implementing mammographic screening programs significantly reduces the incidence of advanced breast cancer, improving early diagnosis and long-term survival rates (32, 33).

In our study, lymph node involvement (N1) was 38.8%; this result is consistent with some reports in the literature that establish a relationship between late diagnosis and the presence of lymphatic involvement, which, in turn, could be an indicator of a worse prognosis (34). However, traditional prognostic factors have also been described as limited in their ability to provide reliable stratification for all patients. Consequently, recent reports have shown that up to 30% of women with lymph node-negative breast cancer die from the disease regardless of adjuvant therapy and 70% survive without adjuvant therapy (35). This finding underscores the importance of a multidisciplinary approach integrating surgery, chemotherapy, and radiotherapy in patients with lymph node involvement to optimize therapeutic outcomes (36).

The absence of metastasis (M0) in 59.7% of cases represents an opportunity for more aggressive therapeutic interventions with radical surgery based on disease assessment. However, this finding also reflects the variability in the stages of breast cancer presentation, suggesting the need to personalize treatment according to the molecular and sociodemographic characteristics of patients, a strategy supported by recent studies in precision oncology (37, 38). Furthermore, the relationship between breast cancer subtypes and TNM staging described in this study underscores the importance of integrating molecular and clinical factors in the diagnosis and characterization of breast cancer, in line with the trend toward personalized treatments that has strengthened in the last decade (39). Notably, treatments that consider both the specific tumor biology and the context of each patient achieve better outcomes and a better quality of life for patients (40).

Our findings also reveal variability in the histological and molecular classification of breast cancer. Regarding intrinsic subtypes, we recorded a significant proportion of “other types” that lack a specific molecular classification (16.4%). This finding is consistent with the understanding that breast cancer is a group of diseases that includes many subtypes with distinctive histology and clinical characteristics. While the approach to some of these subtypes is established, treatment of others is similar to that of the most common types. However, many subtypes lack well-defined clinical guidelines and are diagnosed and treated by extrapolation from the most common cancer types (41). This phenomenon reflects the need for more specific and earlier diagnoses to improve the impact of aggressive subtypes, such as triple-negative, as observed in studies from Mexico (42).

Regarding molecular subtypes, in contrast to our results, a series observed in Colombia describes the predominance of the luminal A subtype, followed by variants of the luminal B subtype. In our cohort, we reported a higher frequency of the luminal B subtype, highlighting variations in the distribution of molecular subtypes and the impact this may have on personalized treatment strategies (43, 44). Similarly, a study conducted in Venezuela observed a high incidence of luminal B and triple-negative subtypes in a population with a younger average age at diagnosis than the one reported in this study. This finding reinforces the importance of accurate molecular classification for the approach to breast cancer (45).

Our findings identify a relationship between histological classification, molecular subtypes, and TNM staging of breast cancer. This fact is of particular importance because this cancer can be present at any clinical stage, and its progression and response to various treatment modalities remain unclear. This means that identifying different molecular subtypes can predict some of these differences (46) and categorize patients into different groups based on the choice of medical and surgical treatment, as well as predict clinical behavior and prognosis (47). In this regard, other studies have found significant correlations between survival status, tumor grade, or size (T), and different histopathological types of breast cancer. However, in contrast to our results, no significant correlations were observed with metastatic disease or TNM stage. Furthermore, none of the correlations between molecular subtypes and age at diagnosis were significant, which is consistent with our results (48).

One of the main limitations of this study is the small sample size, which may affect the generalizability of the results and analyses. Furthermore, the use of convenience sampling introduces the potential for selection bias. Future studies with larger, more representative samples are necessary to validate these findings.

## Conclusion

The study describes the heterogeneity of breast cancer within the analyzed population. Regarding histological type, invasive breast carcinoma of no special type predominates in women over 50 years of age, typically associated with advanced stages and a complicated prognosis. The high frequency of Luminal B, Luminal A, and Triple-Negative subtypes is notable. The highest frequency of identified cases was in the advanced stages, underscoring the need for personalized treatments and improved early detection strategies.

This study serves as a basis for future perspectives related to evaluating the impact of these findings on clinical practice, improving therapeutic decision-making, and personalizing treatment. Future research could focus on the development of artificial intelligence-based predictive tools to improve patient classification and prognosis, as well as longitudinal studies analyzing treatment response according to disease subtype and stage.

## Data Availability

The raw data supporting the conclusions of this article will be made available by the authors, without undue reservation.
